# On the Strong Subregularity of the Optimality Mapping in an Optimal Control Problem with Pointwise Inequality Control Constraints

**DOI:** 10.1007/s00245-022-09959-9

**Published:** 2023-03-13

**Authors:** N. P. Osmolovskii, V. M. Veliov

**Affiliations:** 1 Systems Research Institute, Polish Academy of Sciences, Warsaw, Poland; 2 Institute of Statistics and Mathematical Methods in Economics, TU Wien, Vienna, Austria

**Keywords:** Optimization, Optimal control, Mayer’s problem, Control constraint, metric subregularity, 49K40, 90C31

## Abstract

This paper presents sufficient conditions for strong metric subregularity (SMsR) of the optimality mapping associated with the local Pontryagin maximum principle for Mayer-type optimal control problems with pointwise control constraints given by a finite number of inequalities $G_j(u)\le 0$. It is assumed that all data are twice smooth, and that at each feasible point the gradients $G_j'(u)$ of the active constraints are linearly independent. The main result is that the second-order sufficient optimality condition for a weak local minimum is also sufficient for a version of the SMSR property, which involves two norms in the control space in order to deal with the so-called two-norm-discrepancy.

## Introduction

This paper contributes to the analysis of Lipschitz stability with respect to perturbations of the following Mayer type optimal control problem:

1 $$\begin{aligned}{} & {} \hbox {minimize} \quad J(x,u):=F(x(0),x(1)), \end{aligned}$$

2 $$\begin{aligned}{} & {} \dot{x}(t)=f(x(t),u(t))\quad \hbox {a.e. in}\quad [0,1], \end{aligned}$$

3 $$\begin{aligned}{} & {} G(u(t))\le 0 \quad \hbox {a.e. in}\quad [0,1], \end{aligned}$$

where $F:\mathbb {R}^{2n}\rightarrow \mathbb {R}$, $f: \mathbb {R}^{n+m}\rightarrow \mathbb {R}^n$, and $G:\mathbb {R}^m\rightarrow \mathbb {R}^k$ are of class $C^2$, $u\in L^\infty$, $x\in W^{1,1}$. More precisely, we investigate the property of *Strong Metric subRegularity* (SMsR) of the so-called *optimality mapping*, associated with the system of first order necessary optimality conditions (Pontryagin’s conditions in local form) for problem (1)–(3). These optimality conditions may have various forms. In this paper we deal with the representation using the augmented Hamiltonian, where the control constraints are included with corresponding Lagrange multipliers (see next section for a detailed formulation).

In general, the local Potryagin principle can be written in the form of an inclusion (also called *optimality system*)

$$\begin{aligned} 0 \in \varPhi (y), \end{aligned}$$

where *y* incorporates the state, control, adjoint variables, and possibly the Lagrange multipliers associated with the control constraints. In this general setting, *y* belongs to a metric space $(Y, d_Y)$ and the image of $\varPhi$ is contained in another metric space $(Z, d_Z)$. Each of these spaces is endowed with an additional metric: $d_{Y\!\circ }$ in *Y*, and $d_{Z\circ }$ in *Z*.

The definition of strong metric subregularity of the mapping $\varPhi$ that we use is a slight (however substantial) extension of the standard one, introduced under this name in [9], also see [10, Chapter 3.9] and the recent paper [6]. The difference is, that the definition below involves the four metrics, $d_Y, d_{Y\!\circ }$ in *Y*, and $d_Z, d_{Z\circ }$ in *Z*, instead of a single metric in each of the two spaces.

### Definition 1.1

The set-valued mapping $\varPhi :Y \rightrightarrows Z$ is *strongly metrically subregular* (SMsR) at $(\hat{y}, \hat{z}) \in Y\times Z$ if $\hat{z} \in \Phi (\hat{y})$ and there exist number $\kappa \ge 0$ and neighborhoods $B_Y$ of $\hat{y}$ in the metric $d_{Y\!\circ }$ and $B_Z$ of $\hat{z}$ in the metric $d_{Z\circ }$, such that for any $z \in B_Z$ and any solution $y \in B_Y$ of the inclusion $z \in \varPhi (y)$, it holds that $d_Y(y, \hat{y}) \le \kappa \,d_Z(z, \hat{z})$.

Versions of the SMsR property have also been introduced and utilized in [3, 5, 11]. Metric regularity properties with two norms in the space *Z* (a Banach space) are first introduced in [22], while utilization of two metrics in *Y*, in relation with the SMsR property, is important in [2]. It is well recognized that the SMsR of the optimality mapping in optimal control is a key property for ensuring convergence with error estimates of numerous methods for solving optimal control problems: discretization methods, gradient methods, Newton-type methods, etc. (see e.g. [3, 6, 21], in addition to a large number of papers where the SMsR property is implicitly used).

We mention that there exists an amount of literature on Lipschitz continuity (related to the property of strong metric regularity) and differentiability of the optimal solution with respect to parameters; see e.g. [8] and [13], correspondingly, as well as the bibliography therein. These properties are stronger than SMsR, therefore the corresponding sufficient conditions for their validity are also stronger. On the other hand, the SMsR property is useful enough for the applications mentioned in the last paragraph.

The SMsR property of the optimality mapping associated with optimal control problems has been investigated and used in several papers, e.g. [1, 7, 20, 21]. However, the sufficient conditions obtained in these papers require various kinds of coercivity conditions for a quadratic form defined by the second derivatives of the (augmented) Hamiltonian. These conditions have to be satisfied for *all* (sufficiently small) admissible variations of the reference solution of the optimality system. In the present paper, we require coercivity of this quadratic form on an *extended critical cone* only, which is a subset of the set of all admissible variations. Namely, we establish that the known second-order sufficient optimality conditions for problem (1)–(3) (in terms of the extended critical cone) are also sufficient for SMsR. This makes the conditions for SMsR close to those in mathematical programming. A remarkable additional result is that in the second-order sufficient optimality conditions, the extended critical cone can be replaced with the usual critical cone, provided that a point-wise Legendre-type condition is satisfied. Moreover, we show that the converse is also true: the latter condition together with coercivity of the quadratic form on the critical cone implies coercivity on the extended critical cone.

In Sect. 2 we introduce some basic notations and assumptions. In Sect. 3 we define the extended critical cone and recall a second order sufficient optimality condition ensuring local quadratic growth of the objective function (1). This condition involves coercivity of the quadratic form associated with the Hamiltonian along the directions of the extended critical cone. In Sect. 4 we prove that for the local quadratic growth it suffices to require coercivity on the usual (not extended) critical cone, together with a Legendre-type condition. The main result—the sufficient conditions for SMsR—is formulated in Sect. 5, while the long Sect. 6 contains its proof.

## Notations and Assumptions

First we recall some standard notations. The scalar product and the norm in the Euclidean space $\mathbb {R}^n$ is defined in the usual way: $\langle x, x' \rangle := x_1 x'_1 + \ldots + x_n x'_n$, and $|x| = \sqrt{ \langle x,x \rangle }$ for any $x = (x_1, \ldots , x_n) \in \mathbb {R}^n$ and $x' = (x'_1, \ldots , x'_n) \in \mathbb {R}^n$. The elements of $\mathbb {R}^n$ are regarded as column-vectors with the exception of the adjoint variables *p* and $\lambda$ (to appear later), which are row-vectors. For a function $\psi :\mathbb {R}^k \rightarrow \mathbb {R}^r$ of the variable *z* we denote by $\psi '(z)$ its derivative (Jacobian), represented by an $(r \times k)$-matrix. For $r=1$, $\psi ''(z)$ denotes the second derivative (Hessian), represented by a $(k \times k)$-matrix. For a function $\psi :\mathbb {R}^{k \times q} \rightarrow \mathbb {R}$ of the variables (*z*, *v*), $\psi '(z,v)$ and $\psi ''(z,v)$ still denote the first and the second derivatives with respect to (*z*, *v*), however the partial derivatives are denoted by $\psi _z$, $\psi _v$, $\psi _{zz}$, $\psi _{zv}$ and $\psi _{vv}$.

The space $L^k = L^k([0,1],\mathbb {R}^r)$, with $k = 1, 2$ or $k = \infty$, consists of all (classes of equivalent) Lebesgue measurable *r*-dimensional vector-functions defined on the interval [0, 1], for which the standard norm $\Vert \cdot \Vert _k$ is finite. As usual, $W^{1,1} = W^{1,1}([0,T],\mathbb {R}^r)$ denotes the space of absolutely continuous functions $x:[0,T] \rightarrow \mathbb {R}^r$ for which the first derivative belongs to $L^1$. For convenience, the norm in $W^{1,1}$ is defined as $\Vert x \Vert _{1,1} := |x(0)| + \Vert \dot{x} \Vert _1$, so that $\Vert x \Vert _\infty \le \Vert x \Vert _{1,1}$. The specification $([0,1],\mathbb {R}^r)$ will be omitted if clear from the context.

According to (3), the set of admissible control values is

$$\begin{aligned} U:=\{v\in \mathbb {R}^m: \; G(v)\le 0\}. \end{aligned}$$

Let $G_i$ denote the *i*th component of the vector *G*. For any $v\in U$ define the set of active indices

$$\begin{aligned} I(v)=\{i\in \{1,\ldots ,k\}\,: \quad G_i(v)=0\}. \end{aligned}$$

### Assumption 2.1

*(regularity of the control constraints)* The set *U* is nonempty and at each point $v\in U$ the gradients $G_i'(v)$, $i\in I(v)$ are linearly independent.

In the sequel we use the notation

$$\begin{aligned} q=(x(0),x(1))=(x_0,x_1),\quad w=(x,u), \quad \mathcal{W}=W^{1,1}\times L^\infty . \end{aligned}$$

Similarly, we denote $\hat{w}=(\hat{x},\hat{u})\in \mathcal{W}$, $\hat{q}=(\hat{x}(0),\hat{x}(1))$.

### Assumption 2.2

The triplet $(\hat{w},\hat{p}, \hat{\lambda }) \in \mathcal{W}\times W^{1,1} \times L^\infty$ satisfies the following system of equations and inequalities:

4 $$\begin{aligned}{} & {} \hat{\lambda }(t)\ge 0, \quad \hat{\lambda }(t) G(\hat{u}(t))=0\quad \hbox {a.e. in}\quad [0,1], \end{aligned}$$

5 $$\begin{aligned}{} & {} ( -\hat{p}(0), \hat{p}(1))=F'(\hat{q} ), \end{aligned}$$

6 $$\begin{aligned}{} & {} \dot{\hat{p}}(t) +\hat{p}(t)\, f_x(\hat{w}(t))=0 \quad \hbox {a.e. in}\quad [0,1], \end{aligned}$$

7 $$\begin{aligned}{} & {} \hat{p}(t)\,f_u(\hat{w}(t))+\hat{\lambda }(t) G'(\hat{u}(t))=0 \quad \hbox {a.e. in}\quad [0,1], \end{aligned}$$

8 $$\begin{aligned}{} & {} -\dot{\hat{x}}(t)+f(\hat{w}(t))=0 \quad \hbox {a.e. in}\quad [0,1], \end{aligned}$$

9 $$\begin{aligned}{} & {} G(\hat{u}(t))\le 0 \quad \hbox {a.e. in}\quad [0,1]. \end{aligned}$$

Observe that this system represents the first order necessary optimality condition for a weak local minimum^1^ of the pair $\hat{w}=(\hat{x},\hat{u})$ (see e.g. [14, part 1, section 18]); later on we refer to it as to *optimality system*. Namely, if $\hat{w}$ is a point of weak local minimum in problem (1)–(3), then there exist $\hat{p}\in W^{1,1}$ and $\hat{\lambda }\in L^\infty$ such that the optimality system is fulfilled. Note that for a given $\hat{w}$ the pair $(\hat{p},\hat{\lambda })$ is uniquely determined by these conditions. Indeed, the adjoint variable *p* is uniquely determined by adjoint equation (6) and transversality conditions (5), and then $\hat{\lambda }$ is uniquely determined by equation (7) and complementary slackness condition in (4) due to Assumption 2.1.

Introduce the *Hamiltonian* and the *augmented Hamiltonian *

$$\begin{aligned} H(w,p)=p\, f(w), \quad \bar{H}(w,p,\lambda )=p\, f(w)+\lambda \, G(u). \end{aligned}$$

Then equations (6) and (7) take the form

$$\begin{aligned} -\dot{\hat{p}}(t) = H_x(\hat{w}(t),\hat{p}(t)),\quad \bar{H}_u(\hat{w}(t),\hat{p}(t),\hat{\lambda }(t))=0 \quad \hbox {a.e. in}\quad [0,1]. \end{aligned}$$

Notice that here and below, the dual variables *p* and $\lambda$ are treated as row vectors, while *x*, *u*, *w*, *f*, and *G* are treated as column vectors.

## Second-Order Sufficient Conditions for a Weak Local Minimum

Now we discuss the second-order sufficient conditions for a weak local minimum (references will be given at the end of Sect. 4). Set

$$\begin{aligned} { M}_{j}=\{t\in [0,1]: \; G_j(\hat{u}(t))=0\},\quad j=1,\ldots ,k. \end{aligned}$$

Define the *critical cone*

10 $$\begin{aligned} \begin{array}{rcl} K :=\Big \{\,w\in \mathcal{W}&{}:&{} \dot{x}(t)=f'(\hat{w}(t))w(t), \quad H_u(\hat{w}(t),\hat{p}(t))u(t)=0 \quad \hbox {a.e. in}\quad [0,1],\\ &{}&{} G'_j(\hat{u}(t))u(t)\le 0 \;\;\hbox {a.e. on}\; M_j, \quad j=1,\ldots ,k \,\Big \}. \end{array} \end{aligned}$$

It can be easily verified that $F'(\hat{q} )q =0$ for any element *w* of the critical cone.

Indeed, let $w\in K$. Then $\dot{x}(t)=f'(\hat{w}(t))w(t)$ a.e. in [0, 1]. Multiplying this equation by $\hat{p}(t)$ we get that $\hat{p}(t)\dot{x}(t) =\hat{p}(t)f_x(\hat{w}(t))x(t) + \hat{p}(t)f_u(\hat{w}(t))u(t)$ a.e. in [0, 1]. The equalities $\hat{p}(t)f_x(\hat{w}(t))=-\dot{\hat{p}}(t)$ and $\hat{p}(t)f_u(\hat{w}(t)) u(t)=0$ a.e. in [0, 1], give $\hat{p}(t)\dot{x}(t) + \dot{\hat{p}}(t) x(t)=0$ a.e. in [0, 1]. Integrating this equation on [0, 1], we obtain that $\hat{p}(1)x(1)-\hat{p}(0)x(0) =0$. Using the transversality conditions (5), we get $F_{x_0}(\hat{q})x(0)+F_{x_1}(\hat{q})x(1)=0$ q.e.d.

In many cases (in ”smooth problems” of mathematical programming and the calculus of variations) it is sufficient for local minimality that the critical cone consists only of the zero element. However, this is not the case for optimal control problems with a control constraint of the type $u(t)\in U$.

An equivalent definition of the critical cone is the following. Set

$$\begin{aligned} M^+(\hat{\lambda }_j)=\{t\in [0,1]: \; \hat{\lambda }_j(t)>0\}, \quad j=1,\ldots ,k. \end{aligned}$$

Then, due to (7).

11 $$\begin{aligned} \begin{array}{rcl} K =\Big \{\,w\in \mathcal{W}&{}:&{} \dot{x}(t)=f'(\hat{w}(t))w(t)\quad \hbox {a.e. in}\quad [0,1], \quad G'_j(\hat{u}(t))u(t)\le 0 \;\;\hbox {a.e. on}\; M_j; \\ &{}&{} G'_j(\hat{u}(t))u(t)=0 \;\;\hbox {a.e. on}\; M^+(\hat{\lambda }_j), \quad j=1,\ldots ,k\,\Big \}. \end{array} \end{aligned}$$

We introduce an extension of the critical cone. For any $\Delta >0$ and $j=1,\ldots ,k$ we set

$$\begin{aligned} M^+_\Delta (\hat{\lambda }_j)=\{t\in [0,1]: \; \hat{\lambda }_j(t) >\Delta \}. \end{aligned}$$

For any $\Delta >0$ we set

12 $$\begin{aligned} \begin{array}{rclll} K_\Delta =\Big \{\,w\in W&{}:&{} \dot{x}(t)=f'(\hat{w}(t))w(t)\quad \hbox {a.e. in}\quad [0,1], \quad G'_j(\hat{u}(t))u(t)\le 0 \;\;\hbox {a.e. on}\; M_j, \\ &{}&{} G'_j(\hat{u}(t))u(t)=0 \;\;\hbox {a.e. on}\; M^+_\Delta (\hat{\lambda }_j), \;\; j=1,\ldots ,k \,\Big \}. \end{array} \end{aligned}$$

Notice that the cones $K_\Delta$ form a non-increasing family as $\Delta \rightarrow 0+$. In particular, $K \subset K_\Delta$ for any $\Delta > 0$.

Define the *quadratic form*:

13 $$\begin{aligned} \Omega (w):= & {} \langle F''(\hat{q})q,q\rangle + \int _0^1 \langle \bar{H}_{ww}(\hat{w}(t),\hat{p}(t), \hat{\lambda }(t))w(t),w(t)\rangle \mathrm{\,d}t,\nonumber \\{} & {} \quad \hbox {where} \quad q=(x(0),x(1)). \end{aligned}$$

### Assumption 3.1

There exist $\Delta >0$ and $c_\Delta >0$ such that

14 $$\begin{aligned} \Omega (w)\ge c_\Delta \big (|x(0)|^2 +\Vert u\Vert _2^2\big )\quad \forall \, w\in K_\Delta . \end{aligned}$$

### Remark 3.1

Assumption 3.1 is equivalent to the following: there exist $\Delta >0$ and $c_\Delta >0$ such that

15 $$\begin{aligned} \Omega (w)\ge c_\Delta \big (\Vert x\Vert _\infty ^2 +\Vert u\Vert _2^2\big )\quad \forall \, w\in K_\Delta . \end{aligned}$$

Indeed, if $w\in K_\Delta$, then $\dot{x}(t)=f_x(\hat{w}(t))x(t)+f_u(\hat{w}(t))u(t)$ a.e. in [0, 1], whence

$$\begin{aligned} \Vert x\Vert _\infty \le c(|x(0)|+\Vert u\Vert _1))\le c(|x(0)|+\Vert u\Vert _2) \end{aligned}$$

with some $c>0$. The required equivalence follows.

### Remark 3.2

Notice that if (14) is true for some $\Delta >0$ and $c_\Delta >0$, then it is true for any positive $\Delta '<\Delta$ and the same $c_\Delta$.

In the sequel we use the notations *c*, $c'$, $c''$, $c_1$, $c_2$, etc. for constants which may have different values in different estimations.

We recall the following theorem, first published in [15, 16] in a slightly different formulation.

### Theorem 3.1

(sufficient second order condition) Let Assumptions 2.1, 2.2, and 3.1 be fulfilled. Then there exist $\delta >0$ and $c>0$ such that

16 $$\begin{aligned} J(w)-J(\hat{w})\ge c\big (\Vert x-\hat{x}\Vert _\infty ^2+\Vert u-\hat{u}\Vert ^2_2 \big ) \end{aligned}$$

for all admissible $w=(x,u)\in W^{1,1}\times L^\infty$ such that $\Vert w-\hat{w}\Vert _\infty <\delta$.

In the next section, we discuss the equivalent formulation of this theorem and then provide references to the literature, where proofs can be found.

## An Equivalent Form of the Second-Order Sufficient Condition for Local Optimality

In this section we show that Assumption 3.1 can be reformulated in terms of the critical cone *K*, instead of $K_\Delta$, provided that an additional condition of Legendre type is fulfilled.

Let $(\hat{w},\hat{p}, \hat{\lambda }) \in \mathcal{W}\times W^{1,1} \times L^\infty$, and let Assumptions 2.1 and 2.2 hold.

### Assumption 4.1

There exists $c_0>0$ such that

17 $$\begin{aligned} \Omega (w)\ge c_0\big (|x(0)|^2 +\Vert u\Vert _2^2\big )\quad \forall \, w\in K. \end{aligned}$$

Further, for any $\Delta >0$ and any $t\in [0,1]$ denote by $I\!\!\!\!C_\Delta (t)$ the cone of all vectors $v\in \mathbb {R}^m$ satisfying for all $j=1,\ldots ,k$ the conditions

$$\begin{aligned} \left\{ \begin{array}{l} G_j'(\hat{u}(t))v\le 0 \quad \hbox {if} \quad G_j(\hat{u}(t))=0,\\ G_j'(\hat{u}(t))v=0 \quad \hbox {if} \quad \hat{\lambda }_j(t)>\Delta . \end{array} \right. \end{aligned}$$

For any $\Delta >0$ and any $j\in \{1,\ldots ,k\}$ we set

$$\begin{aligned} m_\Delta (\hat{\lambda }_j) :=\{t\in [0,1] :\; 0<\hat{\lambda }_j(t)\le \Delta \}, \quad m_\Delta : =\bigcup _{j=1}^k m_\Delta (\hat{\lambda }_j). \end{aligned}$$

Clearly, $$\textrm{meas}\, m_\Delta \rightarrow 0$$ as $$\Delta \rightarrow 0+$$.

### Assumption 4.2

*(strengthened Legendre condition on* $m_\Delta$). There exist $\Delta >0$ and $c_{\Delta }^L>0$ such that for a.a. $t\in m_\Delta$ we have

18 $$\begin{aligned} \langle \bar{H}_{uu}(\hat{w}(t),\hat{p}(t), \hat{\lambda }(t)) v, v\rangle \ge c_{\Delta }^L |v|^2\quad \forall \, v\in I\!\!\!\!C_\Delta (t). \end{aligned}$$

### Remark 4.1

Similarly as in Remark 3.2, if (18) is true for some $\Delta >0$ and $c_\Delta ^L>0$, then it is true for any positive $\Delta '<\Delta$ and the same $c_\Delta ^L$.

In the sequel, we often omit the argument *t* of *x*, *u*, $\hat{x}$, $\hat{u}$, etc.

The following lemma follows from the definition of $\Omega$ in (13).

### Lemma 4.1

Let $$w=(x,u)\in \mathcal{W}$$, $$w'=(x',u')\in \mathcal{W}$$. Then

19 $$\begin{aligned} \Omega (w+w')=\Omega (w) + E(w,w'), \end{aligned}$$

where

$$\begin{aligned} E(w,w')= & {} \Omega (w') + 2 \langle F''(\hat{q}) q, q'\rangle \\{} & {} + 2 \int _0^1 \Big (\langle H_{xx}(\hat{w},\hat{p}) x, x'\rangle + \langle H_{xu}(\hat{w},\hat{p}) u, x'\rangle \\{} & {} + \langle H_{ux}(\hat{w},\hat{p}) x, u'\rangle + \langle \bar{H}_{uu}(\hat{w},\hat{p}, \hat{\lambda }) u, u'\rangle \Big )\mathrm{\,d}t \end{aligned}$$

Moreover, there exists a constant *c*, independent of *w* and $w'$, such that

20 $$\begin{aligned}{} & {} \left| E(w,w') - \int _0^1 \langle \bar{H}_{uu}(\hat{w},\hat{p}, \hat{\lambda }) u', u'\rangle \mathrm{\,d}t\right| \nonumber \\{} & {} \quad \le c \left( \Vert x \Vert _\infty \Vert x' \Vert _\infty + \Vert x' \Vert _\infty ^2 + \Vert x' \Vert _\infty \Vert u' \Vert _1\right. \nonumber \\{} & {} \qquad \left. + \Vert x \Vert _\infty \Vert u' \Vert _1 + \Vert x' \Vert _\infty \Vert u \Vert _1 + \Vert \,| u | \cdot | u'| \,\Vert _1\right) . \end{aligned}$$

Henceforth, for $w=(x,u)\in \mathcal{W}$ we set

$$\begin{aligned} \gamma _0( w)=| x(0)|^2+\int _0^1| u|^2\mathrm{\,d}t,\quad \gamma ( w)=\Vert x\Vert ^2_\infty +\int _0^1| u|^2\mathrm{\,d}t. \end{aligned}$$

It is clear that $\gamma _0( w)\le \gamma ( w)$, and, as shown in Remark 3.1, if $\dot{x}=f_w(\hat{w})w$, then there exists $c>0$, independent of *w*, such that

$$\begin{aligned} \gamma (w) \le c \gamma _0(w). \end{aligned}$$

### Proposition 4.1

Assumptions 4.1 and 4.2 imply Assumption 3.1.

### Proof

Let Assumptions 4.1 and 4.2 hold with some $c_0>0$, $\Delta >0$ and $c_{\Delta }^L>0$, where $\Delta$ will be fixed later as small enough, see Remark 4.1. Set

21 $$\begin{aligned} \alpha (\Delta )= \sqrt{\textrm{meas}\, (m_\Delta )}. \end{aligned}$$

Note that $\alpha (\Delta ) \rightarrow 0+$ as $\Delta \rightarrow 0+$. We may assume that $\Delta$ is so small that $\alpha (\Delta )\le 1$.

Let $$\tilde{w} \in K_\Delta $$. Set

$$\begin{aligned} u'= \tilde{u}\chi _{m_\Delta }, \end{aligned}$$

where $\chi _{m_\Delta }$ is the characteristic function of the set $m_\Delta$. Obviously, $u'(t)\in I\!\!\!\!C_\Delta (t)$ a.e. on [0, 1] and, therefore,

$$\begin{aligned} \langle \bar{H}_{uu}(\hat{w}(t),\hat{p}(t), \hat{\lambda }(t)) u'(t), u'(t)\rangle \ge c_{\Delta }^L |u'(t)|^2 \quad \hbox {a.e. on} \quad [0,1]. \end{aligned}$$

Hence,

$$\begin{aligned} \int _0^1 \langle \bar{H}_{uu}(\hat{w},\hat{p}, \hat{\lambda }) u',u'\rangle \mathrm{\,d}t \ge c_{\Delta }^L\int _0^1 |u'|^2\mathrm{\,d}t. \end{aligned}$$

Let $x'$ be the solution to the equation

$$\begin{aligned} \dot{x}'=f_x(\hat{w})x'+ f_u(\hat{w})u',\quad x'(0)=0. \end{aligned}$$

Then

$$\begin{aligned} \Vert x'\Vert _\infty \le c\Vert u'\Vert _1\le c\sqrt{\textrm{meas}\, (m_\Delta )} \Vert u'\Vert _2\le c\,\alpha (\Delta ) \Vert \tilde{u}\Vert _2. \end{aligned}$$

Hence,

$$\begin{aligned} \Vert x'\Vert _\infty \le c\,\alpha (\Delta )\sqrt{\gamma _0(\tilde{w})}, \quad \Vert u'\Vert _1\le \alpha (\Delta ) \sqrt{\gamma _0(\tilde{w})}. \end{aligned}$$

Set

$$\begin{aligned} w'=(x',u'), \quad x=\tilde{x}-x',\quad u=\tilde{u}-u', \quad w=(x,u). \end{aligned}$$

Since $x'(0)=0$, we have

22 $$\begin{aligned} \gamma _0(w')=\int _0^1 |u'|^2\mathrm{\,d}t. \end{aligned}$$

Obviously,

23 $$\begin{aligned} w \in K,\quad \tilde{w}= w+w', \quad |u|\cdot |u'|=0,\quad \gamma _0(\tilde{w})= \gamma _0( w)+\gamma _0( w'). \end{aligned}$$

Using the estimate (20) in Lemma 4.1, Assumptions 4.1, 4.2, and the third relation in (23), we obtain the inequality

24 $$\begin{aligned} \Omega (\tilde{w})\ge & {} c_0 \gamma _0(w) + c_{\Delta }^L \Vert u' \Vert _2^2 \nonumber \\{} & {} - c\left( \Vert x \Vert _\infty \Vert x' \Vert _\infty + \Vert x' \Vert _\infty ^2 + \Vert x' \Vert _\infty \Vert u' \Vert _1 + \Vert x \Vert _\infty \Vert u' \Vert _1 + \Vert x' \Vert _\infty \Vert u \Vert _1\right) .\quad \end{aligned}$$

We consecutively estimate

$$\begin{aligned}{} & {} \Vert x\Vert _\infty \le \Vert \tilde{x}\Vert _\infty +\Vert x'\Vert _\infty \le c\sqrt{\gamma _0(\tilde{w})}+c\,\alpha (\Delta )\sqrt{\gamma _0(\tilde{w})} \le c'\sqrt{\gamma _0(\tilde{w})}, \\{} & {} \Vert x\Vert _\infty \Vert x'\Vert _\infty \le c'' \alpha (\Delta )\gamma _0(\tilde{w}), \\{} & {} \Vert x'\Vert _\infty ^2 \le c^2 \alpha ^2(\Delta )\gamma _0(\tilde{w}), \quad \Vert x'\Vert _\infty \Vert u'\Vert _1\le c \alpha ^2(\Delta ) \gamma _0(\tilde{w}), \\{} & {} \Vert u\Vert _1\Vert x'\Vert _\infty \le \Vert \tilde{u}\Vert _2 \Vert x'\Vert _\infty \le c\, \alpha (\Delta ) \gamma _0(\tilde{w}), \quad \Vert x\Vert _\infty \Vert u'\Vert _1\le c'\, \alpha (\Delta ) \gamma _0(\tilde{w}), \end{aligned}$$

where $c'$ and $c''$ are appropriate constants. Using these relations and (22) in (24), we obtain that

$$\begin{aligned} \Omega (\tilde{w}) \ge c_0 \gamma _0(w) + c_\Delta ^L \gamma _0(w') - c''' {\alpha }(\Delta ) \gamma _0(\tilde{w}). \end{aligned}$$

with some constant $c'''$. Take $\Delta >0$ such that

$$\begin{aligned} c_\Delta :=\min \{c_0,c_{\Delta }^L\} - c'''\alpha (\Delta )>0, \end{aligned}$$

keeping the same constant $c_{\Delta }^L$ (see Remark 4.1). Then

$$\begin{aligned} \Omega (\tilde{w})\ge c_\Delta \gamma _0(\tilde{w}), \end{aligned}$$

which completes the proof, since $c_\Delta$ is independent of $\tilde{w} \in K_{\Delta }$. $\square$

The converse is also true.

### Proposition 4.2

Assumption 3.1 implies Assumptions 4.1 and 4.2.

### Proof

Let Assumption 3.1 be fulfilled, i.e., there exist $\Delta >0$ and $c_\Delta >0$ such that

$$\begin{aligned} \Omega (w)\ge c_\Delta \gamma _0(w)\quad \forall \, w\in K_\Delta . \end{aligned}$$

According to Remark 3.2, one may fix $\Delta > 0$ arbitrarily small without changing $c_\Delta$, which will be done below.

Since $K\subset K_\Delta$, this inequality holds also on *K*, therefore Assumption  4.1 is fulfilled.

Let us prove that Assumption 4.2 is also fulfilled. Take any $u\in L^\infty$ satisfying the conditions

25 $$\begin{aligned} u(t)\in I\!\!\!\!C_\Delta (t) \quad \hbox {a.e. on}\quad m_\Delta , \quad u\chi _{m_\Delta }=u, \end{aligned}$$

where $\chi _{m_\Delta }$ is the characteristic function of the set $m_\Delta$. Define *x* by the conditions

$$\begin{aligned} \dot{x}=f_x(\hat{w})x+ f_u(\hat{w})u,\quad x(0)=0. \end{aligned}$$

Set $w=(x,u)$. Then, obviously, $w\in K_\Delta$, whence it follows that

$$\begin{aligned} \Omega (w)\ge c_\Delta \gamma _0(w), \quad \hbox {where}\quad \gamma _0(w)=\int _0^1 |u|^2\mathrm{\,d}t. \end{aligned}$$

Moreover,

$$\begin{aligned} \Vert x\Vert _\infty \le c\Vert u\Vert _1\le c\sqrt{\textrm{meas}\, (m_\Delta )}\Vert u\Vert _2= c\,\alpha (\Delta )\sqrt{\gamma _0(w)}, \end{aligned}$$

where $\alpha (\Delta )$ is defined in (21). The latter implies that

$$\begin{aligned}{} & {} |\langle F''(\hat{q})q,q\rangle |\le c' \alpha ^2(\Delta ) \gamma _0(w), \\{} & {} \Vert \langle \bar{H}_{xx}(\hat{w},\hat{p}, \hat{\lambda }) x, x\rangle + 2 \langle \bar{H}_{xu}(\hat{w},\hat{p}, \hat{\lambda }) u, x\rangle \Vert _1\le c' \alpha ^2(\Delta ) \gamma _0(w) \end{aligned}$$

with some $c'>0$. Using these estimates and (13), we get

$$\begin{aligned} 2 c' \alpha ^2(\Delta ) \gamma _0(w)+ \int _0^1 \langle \bar{H}_{uu}(\hat{w},\hat{p}, \hat{\lambda }) u, u\rangle \mathrm{\,d}t\ge \Omega (w)\ge c_\Delta \gamma _0(w). \end{aligned}$$

Take any $\Delta >0$ such that

$$\begin{aligned} c_{\Delta }^L:= -2c'\alpha ^2(\Delta ) + c_\Delta >0. \end{aligned}$$

Then we have

$$\begin{aligned} \int _0^1 \langle \bar{H}_{uu}(\hat{w},\hat{p}, \hat{\lambda }) u, u\rangle \mathrm{\,d}t\ge c_{\Delta }^L \int _0^1 |u|^2\mathrm{\,d}t. \end{aligned}$$

This inequality holds for any $u\in L^\infty$ satisfying (25). The strengthened Legendre condition on $m_\Delta$ follows. $\square$

Thus, instead of Assumption 3.1 we can use Assumptions 4.1 and 4.2 in the sufficient second-order conditions of Theorem 3.1.

The connection between the strengthened Legendre condition and the so-called “local quadratic growth of the Hamiltonian” (defined below) was studied in [4]. Let us formulate the corresponding result from [4] which may be useful for the problem under consideration.

### Definition 4.1

We say that the local quadratic growth condition of the Hamiltonian is fulfilled if there exist $c_H>0$, $\delta >0$ and $\Delta >0$ such that for a.a. $t\in m_\Delta$ we have

$$\begin{aligned} H(\hat{x}(t),u,\hat{p}(t))-H(\hat{x}(t),\hat{u}(t),\hat{p}(t))\ge c_H|u-\hat{u}(t)|^2 \end{aligned}$$

for all $u\in \mathbb {R}^m$ such that $G(u)\le 0$ and $|u-\hat{u}(t)| <\delta$.

### Proposition 4.3

[4] Assumption 4.2 implies the local quadratic growth condition of the Hamiltonian.

The converse is not true. As shown in [4], the condition of the local quadratic growth of the Hamiltonian is somewhat finer than Assumption 4.2.

There is the following more subtle second-order sufficient condition for a weak local minimum at the point $\hat{w}$ in problem (1)–(3).

### Theorem 4.1

(sufficient second order condition) Let Assumptions 2.1, 2.2, and 4.1 hold and the local quadratic growth condition of the Hamiltonian be satisfied. Then there exist $\delta >0$ and $c>0$ such that

26 $$\begin{aligned} J(w)-J(\hat{w})\ge c\big (\Vert x-\hat{x}\Vert _\infty ^2+\Vert u-\hat{u}\Vert ^2_2 \big ) \end{aligned}$$

for all admissible $w=(x,u)\in W^{1,1}\times L^\infty$ such that $\Vert w-\hat{w}\Vert _\infty <\delta$.

A sufficient second order condition of this type for a much more general optimal control problem (together with the corresponding second order necessary condition) was first published by the first author back in 1978 in [12]. A relatively simple proof of Theorem 4.1 in the case of $k=1$ was recently published in [19]. Proofs of much more general results of this type can be found, for example, in [17] and [18].

## Strong Metric Subregularity

In this section we formulate the main result in this paper. Namely, we prove that the optimality mapping associated with problem (1)–(3) is strongly metrically subregular at a reference solution $(\hat{w},\hat{p},\hat{\lambda }) = (\hat{x},\hat{u},\hat{p},\hat{\lambda }) \in \mathcal{W}\times W^{1,1} \times L^\infty$ of the optimality system (4)–(9), provided that Assumptions 2.1, 2.2 and 3.1 hold.

In the sequel, for $w=(x,u)\in \mathcal{W}$ we set

$$\begin{aligned} \Delta w=w-\hat{w},\quad \gamma (\Delta w)=\Vert \Delta x\Vert _\infty ^2+\Vert \Delta u\Vert _2^2. \end{aligned}$$

Consider the *perturbed system * of optimality conditions (4)–(9):

27 $$\begin{aligned}{} & {} \lambda \ge 0, \quad \lambda (G( u)-\eta )=0, \end{aligned}$$

28 $$\begin{aligned}{} & {} (-p(0), p(1))= F'(q)+\nu , \end{aligned}$$

29 $$\begin{aligned}{} & {} \dot{p} + p \,f_x(w)= \pi , \end{aligned}$$

30 $$\begin{aligned}{} & {} pf_u(w)+\lambda G'(u)=\rho , \end{aligned}$$

31 $$\begin{aligned}{} & {} - \dot{x} + f(x,u) =\xi \end{aligned}$$

32 $$\begin{aligned}{} & {} G(u)\le \eta , \end{aligned}$$

where $p \in W^{1,1}$, $\lambda \in L^\infty$, $\nu \in \mathbb {R}^{2n}$, $\pi \in L^1$, $\rho \in L^\infty$, $\xi \in L^1$, $\eta \in L^\infty$. Note that $\nu$, $\pi$, and $\rho$ are treated as row vectors, while $\xi$ and $\eta$ are treated as column vectors. Below we set

33 $$\begin{aligned}{} & {} \Delta x=x-\hat{x},\quad \Delta u=u-\hat{u},\quad \Delta w=(\Delta x,\Delta u)= w-\hat{w},\quad \Delta p=p-\hat{p},\nonumber \\{} & {} \quad \Delta \lambda =\lambda -\hat{\lambda }, \nonumber \\{} & {} \Delta q=(\Delta x(0),\Delta x(1))= (x(0)-\hat{x}(0), x(1)-\hat{x}(1))= (\Delta x_0, \Delta x_1),\nonumber \\{} & {} \omega =(\nu ,\pi , \rho ,\xi , \eta ), \quad \Vert \omega \Vert :=|\nu |+ \Vert \pi \Vert _1+\Vert \rho \Vert _2+ \Vert \xi \Vert _1+\Vert \eta \Vert _2. \end{aligned}$$

### Theorem 5.1

Let Assumptions 2.1, 2.2, and 3.1 be fulfilled. Then there exist reals $\delta >0$ and $\kappa > 0$ such that if

34 $$\begin{aligned} |\nu |+\Vert \pi \Vert _1+ \Vert \rho \Vert _\infty + \Vert \xi \Vert _1 + \Vert \eta \Vert _\infty \le \delta , \end{aligned}$$

then for any solution $(x,u,p,\lambda )$ of the perturbed system (27)–(32) such that $\Vert \Delta w\Vert _\infty \le \delta$ the following estimates hold:

$$\begin{aligned} \Vert \Delta x\Vert _{1,1}\le \kappa \Vert \omega \Vert ,\quad \Vert \Delta u\Vert _2 \le \kappa \Vert \omega \Vert , \\ \Vert \Delta p\Vert _{1,1} \le \kappa \Vert \omega \Vert , \quad \Vert \Delta \lambda \Vert _2\le \kappa \Vert \omega \Vert . \end{aligned}$$

Observe that if the disturbance $\eta$ is not present in the disturbed optimality system (27)–(32), that is, $\eta = 0$, then the inequality (34) follows (modulo a multiplicative constant) from the assumption $\Vert \Delta w \Vert _\infty \le \delta$, together with the equations (28)–(31). Therefore, the claim of the theorem in this case is valid without assuming (34). In this case again, two metrics are needed in Definition 1.1 of SMsR only in the space $Y := W^{1,1} \times L^\infty \times W^{1,1} \times L^\infty$. The neighborhood $B_Y$ in Definition 1.1 is $B_Y := \{ (w,p,\lambda ) \, :\;\Vert w - \hat{w}\Vert _\infty \le \delta \}$ while the metric $d_Y$ is induced by the norm $\Vert (w,p,\lambda ) \Vert := \Vert x\Vert _{1,1} + \Vert p\Vert _{1,1} + \Vert u\Vert _2 + \Vert \lambda \Vert _2$. The metric in *Z* is induced by the norm $\Vert \omega \Vert$ in (33).

## Proof of Theorem 5.1

**1.** We start with the following auxiliary statement related to the constraint $G(u)\le 0.$ Let

$$\begin{aligned} I=\{i_1,\ldots , i_s\}\subset \{1,\ldots ,k\} \end{aligned}$$

be a nonempty set of indices, and let $G_I(v)$ be a column vector with elements $G_{i_1}(v), \ldots , G_{i_s}(v)$. Set

$$\begin{aligned} A_I(v)=G_I'(v)(G_I'(v))^*,\quad \mu _I(v)=|\det A_I(v)|,\quad Q_I=\{v\in B:\;G_I(v)=0\}, \end{aligned}$$

where *B* is a fixed closed ball in $\mathbb {R}^m$. Then, according to Assumption 2.1,

$$\begin{aligned} \mu _I(v)>0 \quad \hbox {for all} \quad v\in Q_I. \end{aligned}$$

For any $\varepsilon >0$, we set

$$\begin{aligned} Q_{I,\varepsilon }=\{v\in B:\;|G_i(v)|\le \varepsilon \quad \hbox {for all}\quad i\in I\,\}. \end{aligned}$$

### Lemma 6.1

There exist positive numbers $\hat{c}$ and $\hat{\varepsilon }$ such that

$$\begin{aligned} \mu _I(v)\ge \hat{c} \quad \hbox {for all} \;\; I \subset \{1, \ldots , k \} \;\; \hbox {and for all} \;\; v\in Q_{I,\hat{\varepsilon }}. \end{aligned}$$

### Proof

Since there are finite number of subsets $I \in \{ 1, \ldots , k\}$, it is enough to prove the lemma for a fixed *I*. If the statement is false, then there exists a sequence $v_s \in B$ such that $G_I(v_s) \rightarrow 0$ with $s \rightarrow \infty$ and $\mu _I(v_s) \le s^{-1}$. Without loss of generality we assume that $v_s$ converges to some vector $v\in B$. Then $G_I(v)=0$ and $\mu _I(v)=0$. A contradiction. $\square$

Since *G* is uniformly continuous on the compact set *B*, there exists $\hat{\delta }>0$ such that

35 $$\begin{aligned} |G(v)-G(v')|\le \hat{\varepsilon }\quad \hbox { whenever}\quad v,v'\in B \quad \hbox { and} \quad |v-v'|\le \hat{\delta }. \end{aligned}$$

Decreasing, if necessary, $\hat{\delta }$, we can assume that $\hat{\delta }\le \hat{\varepsilon }$.

**2.** We analyze conditions (27)–(32). Take any $\delta >0$ such that $\delta \le \hat{\delta }$. Suppose that a collection $(\nu ,\pi ,\rho ,\xi ,\eta )$ satisfies condition (34) and there exists a solution $(x,u,p,\lambda )$ of the perturbed system (27)–(32) such that $\Vert \Delta w\Vert _\infty \le \delta$. Consider this solution. It is clear, that $\Vert w\Vert _\infty$ is bounded (that is, $\Vert w\Vert _\infty \le C$, where $C>0$ does not depend on *w*), and $\Vert \omega \Vert \le \delta$.

Further, note that $\Vert p\Vert _{1,1}$ is bounded due to conditions (28) and (29) and also because $\Vert w\Vert _\infty$, $|\nu |$ and $\Vert \pi \Vert _1$ are bounded. Therefore, $\Vert \Delta p\Vert _{1,1}$ is also bounded. Moreover, the following is true.

### Proposition 6.1

The norms $\Vert \lambda \Vert _\infty$ and $\Vert \Delta \lambda \Vert _\infty$ are bounded.

### Proof

For the ball appearing in Part 1 of the proof we choose $B := \{v \in \mathbb {R}^m \, :\;|v| \le \Vert \hat{u} \Vert _\infty + \delta \}$. Consider equation (30):

$$\begin{aligned} p(t)f_u(w(t))+\lambda (t) G'(u(t))=\rho (t)\quad \hbox {for a.a.}\quad t\in [0,1]. \end{aligned}$$

We assume that $\lambda \ne 0$, otherwise the claims of the proposition are obvious. Set

$$\begin{aligned} M(\lambda )=\{t\in [0,1]: \quad \lambda (t)\ne 0\}. \end{aligned}$$

Then $\textrm{meas}\, M(\lambda )>0$. For any $t\in M(\lambda )$ we set

$$\begin{aligned} I(t)=\{i\in \{1,\ldots , k\}: \; {\lambda }_i(t)>0\}, \quad \lambda _{I(t)}(t)= \{\lambda _{i}(t) \}_{ i\in I(t) }. \end{aligned}$$

Let $t\in M(\lambda )$. The complementary slackness conditions

$$\begin{aligned} \lambda _i(t)\big (G_i(u(t))-\eta _i(t)\big )=0,\quad i=1,\ldots , k, \end{aligned}$$

imply that $G_i(u(t))-\eta _i(t)=0$ for all $i\in I(t)$, and then, $|G_i(u(t))|=|\eta _i(t)|$ for all $i\in I(t)$. Therefore, in virtue of (34),

$$\begin{aligned} |G_{I(t)}(u(t))|\le |\eta (t)|\le \delta . \end{aligned}$$

Since $\delta \le \hat{\delta }$, we obtain

$$\begin{aligned} u(t)\in Q_{I(t),\hat{\delta }}\quad \hbox {for a.a.} \quad t\in M(\lambda ). \end{aligned}$$

Here $G_{I(t)}$ and $Q_{I(t),\hat{\delta }}$ are defined similarly to $G_{I}$ and $Q_{I,\hat{\delta }}$ in Part 1 of the proof. Hence, by Lemma 6.1, and since $\hat{\delta }\le \hat{\varepsilon }$,

$$\begin{aligned} |\det A_{I(t)}(u(t)))| \ge \hat{c}>0 \quad \hbox {for a.a.}\quad t\in M(\lambda ), \end{aligned}$$

where

$$\begin{aligned} A_{I(t)}(u(t))= G'_{I(t)}(u(t))(G'_{I(t)}(u(t)))^*. \end{aligned}$$

Obviously, $\lambda (t) G'(u(t))= \lambda _{I(t)}(t)G'_{I(t)}(u(t))$ for a.a. $t\in M(\lambda )$, and, therefore,

$$\begin{aligned} p(t)f_u(w(t))+\lambda _{I(t)}(t)G'_{I(t)}(u(t))=\rho (t)\quad \hbox {for a.a.}\quad t\in M(\lambda ). \end{aligned}$$

(Note that the dimensions of the vector $$\lambda _{I(t)}(t)$$ and the matrices $$G'_{I(t)}(u(t))$$ and $$ A_{I(t)}(u(t))$$ depend on *t*.) Multiplying this equation by the transposed matrix $$(G'_{I(t)}(u(t)))^*$$ on the right, we get

$$\begin{aligned}{} & {} p(t)f_u(w(t))(G'_{I(t)}(u(t)))^*+ \lambda _{I(t)}(t)A_{I(t)}(u(t)))\nonumber \\{} & {} \quad = \rho (t)(G'_{I(t)}(u(t)))^*\quad \hbox {for a.a.}\quad t\in M(\lambda ). \end{aligned}$$

Then

$$\begin{aligned}{} & {} p(t)f_u(w(t))(G'_{I(t)}(u(t)))^*(A_{I(t)}(u(t)))^{-1}+ \lambda _{I(t)}(t)\\{} & {} \quad =\rho (t)(G'_{I(t)}(u(t)))^*(A_{I(t)}(u(t)))^{-1} \end{aligned}$$

for a.a. $t\in M(\lambda )$. Since here all matrices are essentially bounded and $|\lambda (t)|=|\lambda _{I(t)}(t)|$ for a.a. $t\in M(\lambda )$, we obtain the estimate

$$\begin{aligned} |\lambda (t)|\le C\big (|p(t)|+|\rho (t)| \big ) \quad \hbox {for a.a.}\quad t\in M(\lambda ) \end{aligned}$$

with some $C>0$, and therefore,

$$\begin{aligned} \Vert \lambda \Vert _\infty \le C(\Vert p\Vert _\infty +\Vert \rho \Vert _\infty ). \end{aligned}$$

Since $\Vert p\Vert _\infty$ is bounded and $\Vert \rho \Vert _\infty \le \delta$, we obtain that $\Vert \lambda \Vert _\infty$ is bounded. Hence $\Vert \Delta \lambda \Vert _\infty$ is also bounded. $\square$

**3.** Further, subtracting (8) from (31) we obtain that

36 $$\begin{aligned} -\Delta \dot{x}+f(w)-f(\hat{w})=\xi . \end{aligned}$$

It follows that

$$\begin{aligned} |\Delta x(t)|\le |\Delta x_0|+\Vert \xi \Vert _1+L\Vert \Delta u\Vert _1 + L\int _{0}^t|\Delta x(\tau )|\mathrm{\,d}\tau , \quad t\in [0,1], \end{aligned}$$

with some $L>0$, where

$$\begin{aligned} \Delta x_0=\Delta x(0). \end{aligned}$$

Using the Grönwall inequality, we get

37 $$\begin{aligned} \Vert \Delta x\Vert _{1,1}\le C\big (|\Delta x_0| +\Vert \Delta u\Vert _1+\Vert \xi \Vert _1\big ) \end{aligned}$$

with some $C>0$. In what follows we use a more rough estimate. Namely, since $\Vert \Delta u\Vert _1\le \Vert \Delta u\Vert _2$ and $\Vert \xi \Vert _1\le \Vert \omega \Vert$, we have

38 $$\begin{aligned} \Vert \Delta x\Vert _{1,1}\le C\big (|\Delta x_0| +\Vert \Delta u\Vert _2+\Vert \omega \Vert \big ). \end{aligned}$$

Consequently,

39 $$\begin{aligned} |\Delta q|\le 2C\big (|\Delta x_0| +\Vert \Delta u\Vert _2+\Vert \omega \Vert \big ). \end{aligned}$$

Clearly, relation (36) implies

40 $$\begin{aligned} -\Delta \dot{x}+f'(\hat{w})\Delta w +O(|\Delta w|^2)=\xi . \end{aligned}$$

As usual, for $\varepsilon \in \mathbb {R}_+$, the symbol $O(\varepsilon )$ means that there exists a constant $C>0$, independent of $\varepsilon$, such that $|O(\varepsilon )|\le C|\varepsilon |$ as $\varepsilon \rightarrow 0+$, and the symbol $o(\varepsilon )$ means that $o(\varepsilon )/\varepsilon \rightarrow 0$ as $\varepsilon \rightarrow 0+$. We use these symbols for $O(\varepsilon )$ and $o(\varepsilon )$, taking values in $\mathbb {R}$ or in $\mathbb {R}^n$. Moreover, throughout the paper, the functions *O* and *o* may directly depend on $\Delta w$, not only on the norms appearing as arguments at the place of $\varepsilon$. However, the “smallness” with respect to the arguments of *O* and *o* will be uniform in $\Delta w$, satisfying $\Vert \Delta w \Vert _\infty \le \delta$. For example, $O(|\Delta w|^2)$ in (40), which is a shortening of $O(|\Delta w(t)|^2)$, means that there exists a constant *C* such that $O(|\Delta w(t)|^2) \le C |\Delta w(t)|^2$ for all $\Delta w$ satisfying $\Vert \Delta w \Vert _\infty \le \delta$ and for a.e. $t \in [0,1]$. Similarly, $o(\gamma (\Delta w))$, appearing later, means that $o(\gamma (\Delta w))/\gamma (\Delta w) \rightarrow 0$ with $\gamma (\Delta w) \rightarrow 0$, uniformly with respect $\Delta w$ satisfying $\Vert \Delta w \Vert _\infty \le \delta$.

**4.** Subtracting (5) from (28) we obtain

$$\begin{aligned} (-\Delta p(0), \Delta p(1))= F'(q)-F'(\hat{q})+\nu , \end{aligned}$$

hence,

41 $$\begin{aligned} (-\Delta p(0), \Delta p(1))= F''(\hat{q}) \Delta q + o( |\Delta q|) +\nu . \end{aligned}$$

This implies that

42 $$\begin{aligned} | \Delta p(0)|+ | \Delta p(1)| \le C\big ( |\Delta q|+|\nu |\big ) \end{aligned}$$

with some $C>0$. Multiplying (41) by $\Delta q=(\Delta x(0),\Delta x(1))$, we obtain

43 $$\begin{aligned} \Delta p\Delta x\mid _0^1= \langle F''(\hat{q}) \Delta q,\Delta q\rangle + o( |\Delta q|^2) +\nu \Delta q. \end{aligned}$$

**5.** Subtracting (6) from (29) we obtain

44 $$\begin{aligned} \Delta \dot{p} + p \,f_x(w)-\hat{p} f_x(\hat{w})= \pi . \end{aligned}$$

Using the Grönwall inequality and the inequality $\Vert \Delta u\Vert _1\le \Vert \Delta u\Vert _2$ we get

45 $$\begin{aligned} \Vert \Delta p\Vert _{1,1} \le c\big ( | \Delta p(0)| +\Vert \Delta x\Vert _\infty +\Vert \Delta u\Vert _2 + \Vert \pi \Vert _1\big ) \end{aligned}$$

with some $c>0$. Using (38), (39), (42) in this inequality, and also taking into account the definition of $\Vert \omega \Vert$, we obtain

46 $$\begin{aligned} \Vert \Delta p\Vert _{1,1} \le C\big ( | \Delta x_0|+\Vert \Delta u\Vert _2+\Vert \omega \Vert \big ) \end{aligned}$$

with some $C>0$. Moreover, since $\Vert \Delta w\Vert _\infty \le \delta$ and $\Vert \omega \Vert \le \delta$, we also get

47 $$\begin{aligned} \Vert \Delta p\Vert _{1,1} \le 2C\delta . \end{aligned}$$

Further, we have

$$\begin{aligned}{} & {} p \,f_x(w)-\hat{p} f_x(\hat{w})=\hat{p}(f_x(w) - f_x(\hat{w}))+\Delta pf_x(w) \\{} & {} \quad =\hat{p} f_{xw}(\hat{w})\Delta w+\Delta pf_x(\hat{w}) +\Delta pf_{xw}(\hat{w}) \Delta w +o(|\Delta w|) \\{} & {} \quad = H_{xw}(\hat{w},\hat{p})\Delta w +\Delta pf_x(\hat{w}) +\Delta pf_{xw}(\hat{w}) \Delta w +o(|\Delta w|). \end{aligned}$$

Therefore, relation (44) implies

48 $$\begin{aligned} \Delta \dot{p}+ H_{xw}(\hat{w},\hat{p})\Delta w + \Delta pf_x(\hat{w}) +\Delta pf_{xw}(\hat{w}) \Delta w +o(|\Delta w|) = \pi . \end{aligned}$$

**6.** Next we analyze condition (30). Subtracting (7) from (30), we obtain

$$\begin{aligned} pf_u(w)- \hat{p}f_u(\hat{w})+\lambda G'(u)-\hat{\lambda }G'(\hat{u})=\rho . \end{aligned}$$

Consequently,

$$\begin{aligned} \hat{p}(f_u(w)- f_u(\hat{w})) +\Delta p f_u(w)+ \hat{\lambda }(G'(u)- G'(\hat{u})) +\Delta \lambda G'(u) =\rho . \end{aligned}$$

From here

$$\begin{aligned} \hat{p} f_{uw}(\hat{w})\Delta w +\Delta p f_u(\hat{w}) +\Delta p f_{uw}(\hat{w})\Delta w+ \hat{\lambda }G''(\hat{u})\Delta u +\Delta \lambda G'( u) +o(|\Delta w|)=\rho . \end{aligned}$$

Here,

$$\begin{aligned} \hat{p} f_{uw}(\hat{w})\Delta w= H_{uw}(\hat{w},\hat{p})\Delta w = H_{ux}(\hat{w},\hat{p})\Delta x + H_{uu}(\hat{w},\hat{p})\Delta u. \end{aligned}$$

Therefore,

$$\begin{aligned}{} & {} H_{ux}(\hat{w},\hat{p})\Delta x + H_{uu}(\hat{w},\hat{p})\Delta u + \Delta p f_u(\hat{w}) +\Delta p f_{uw}(\hat{w})\Delta w \\{} & {} \quad + \hat{\lambda }G''(\hat{u})\Delta u +\Delta \lambda G'( u) +o(|\Delta w|) = \rho . \end{aligned}$$

Since $$\bar{H}=H+\lambda G$$,

49 $$\begin{aligned} H_{ux}(\hat{w},\hat{p})\Delta x+ & {} \bar{H}_{uu}(\hat{w},\hat{p},\hat{\lambda })\Delta u + \Delta p f_u(\hat{w}) +\Delta p f_{uw}(\hat{w})\Delta w + \Delta \lambda G'( u)\nonumber \\+ & {} o(|\Delta w|)=\rho . \end{aligned}$$

Using this equality and the boundedness of $\Vert \Delta \lambda \Vert _\infty$ and $\Vert \Delta w\Vert _\infty$, we estimate

50 $$\begin{aligned} |\Delta \lambda G'( u)|\le C\big (|\Delta x| +|\Delta u|+|\Delta p|+|\rho |\big ) \end{aligned}$$

with some $$C>0$$.

In the next paragraphs, we shall utilize Assumption 2.1 and Lemma 6.1 to estimate for a.e $t \in [0,1]$

51 $$\begin{aligned} |\Delta \lambda |\le C'\big (|\Delta x| +|\Delta u|+|\Delta p|+|\rho |\big ). \end{aligned}$$

with some $$C'>0$$.

Set

$$\begin{aligned} M(\Delta \lambda )=\{t\in [0,1]:\quad \Delta \lambda (t)\ne 0 \}. \end{aligned}$$

If $\textrm{meas}\, M(\Delta \lambda ) = 0$ the estimate is trivial, therefore we assume that $\textrm{meas}\, M(\Delta \lambda )>0$. For any $t\in M(\Delta \lambda )$, we set

$$\begin{aligned} J(t)=\{j\in \{1,\ldots ,k\}: \quad \Delta \lambda _j(t)\ne 0 \}. \end{aligned}$$

Let $\Delta \lambda _{J(t)}(t)$ be a row vector, composed of all nonzero components of $\Delta \lambda (t)$, and let $G_{J(t)}$ be a column vector with the components $G_j$ for all $j\in J(t)$. Then, obviously,

52 $$\begin{aligned} | \Delta \lambda (t)|=|\Delta \lambda _{J(t)}(t)|,\quad \Delta \lambda (t) G'( u(t)) =\Delta \lambda _{J(t)}(t) G'_{J(t)}( u(t)) \quad \hbox {for a.a.}\quad t \in M(\Delta \lambda ).\nonumber \\ \end{aligned}$$

Let $t \in M(\Delta \lambda )$, $j\in J(t)$. If $\lambda _j(t)>0$, then, by the complementary slackness condition in (27), we have $G_j(u(t))=\eta _j(t)$, and hence, $|G_j(u(t))| \le \hat{\varepsilon }$ since $\Vert \eta \Vert _\infty \le \delta \le \hat{\delta }\le \hat{\varepsilon }$.

If $\lambda _j(t)=0$, then $\hat{\lambda }_j(t)>0$, and then, by the complementary slackness condition in (4), we have $G_j(\hat{u}(t))=0$. But then, since $\Vert u-\hat{u}\Vert _\infty \le \hat{\delta }$, by condition (35) we again have $|G_j(u(t))| \le \hat{\varepsilon }$.

Thus, for all $j\in J(t)$ we have $|G_j(u(t))| \le \hat{\varepsilon }$. This implies that

$$\begin{aligned} u(t)\in Q_{J(t), \hat{\varepsilon }} \quad \hbox {for a.a.}\quad t\in M(\Delta \lambda ), \end{aligned}$$

where the set $Q_{J(t),\hat{\varepsilon }}$ is defined similarly to the set $Q_{I,\varepsilon }$ and the ball *B* is defined as at the beginning of the proof of Proposition 6.1. By Lemma 6.1, it follows that

$$\begin{aligned} |\det A_{J(t)}(u(t))| \ge \hat{c}>0 \quad \hbox {for a.a.}\quad t\in M(\Delta \lambda ), \end{aligned}$$

where

$$\begin{aligned} A_{J(t)}(u(t))= G'_{J(t)}(u(t))(G'_{J(t)}(u(t)))^*. \end{aligned}$$

Let

$$\begin{aligned} z(t):= \Delta \lambda (t) G'( u(t)), \quad t\in [0,1]. \end{aligned}$$

According to (50) and the second equality in (52) we have

53 $$\begin{aligned} |z(t)|\le C\big (|\Delta x(t)| +|\Delta u(t)|+|\Delta p(t)|+|\rho (t)|\big ), \quad z(t)= \Delta \lambda _{J(t)}(t) G'_{J(t)}( u(t))\nonumber \\ \end{aligned}$$

for a.a. $$t\in M(\Delta \lambda )$$. Consequently,

$$\begin{aligned} z(t)(G'_{J(t)}( u(t)))^*=\Delta \lambda _{J(t)}(t) A_{J(t)}(u(t)), \end{aligned}$$

hence,

$$\begin{aligned} z(t)(G'_{J(t)}( u(t)))^*A^{-1}_{J(t)}(u(t))=\Delta \lambda _{J(t)}(t). \end{aligned}$$

This equality, the inequality in (53), and the equality $|\Delta \lambda (t)|=|\Delta \lambda _{J(t)}(t)|$, satisfied for a.a. $t\in M(\Delta \lambda )$, imply estimate (51).

Estimate (51) together with the inequalities $\Vert \Delta w\Vert _\infty \le \delta$, (34), and (47) imply

54 $$\begin{aligned} \Vert \Delta \lambda \Vert _\infty \le C\delta \end{aligned}$$

with some $C>0$. In addition, from (38), (46), and (51) it follows that

55 $$\begin{aligned} \Vert \Delta \lambda \Vert _2\le C\big (|\Delta x_0|+\Vert \Delta u\Vert _2 +\Vert \omega \Vert \big ) \end{aligned}$$

with some $$C>0$$.

**7.** Next, we estimate $\Omega (\Delta w)$. Multiplying (48) by $\Delta x$, we get

56 $$\begin{aligned}{} & {} \Delta \dot{p}\,\Delta x +\langle H_{xw}(\hat{w},\hat{p})\Delta w,\Delta x\rangle +\Delta pf_x(\hat{w})\Delta x +\langle \Delta pf_{xw}(\hat{w}) \Delta w,\Delta x\rangle +o(|\Delta w|^2)\nonumber \\{} & {} \quad = \pi \Delta x. \end{aligned}$$

Further, since

$$\begin{aligned} G'(u)=G'(\hat{u})+ G''(\hat{u})\Delta u+o(|\Delta u|) \end{aligned}$$

and $\Vert \Delta \lambda \Vert _\infty$ is bounded, relation (49) implies

$$\begin{aligned}{} & {} H_{ux}(\hat{w},\hat{p})\Delta x + \bar{H}_{uu}(\hat{w},\hat{p},\hat{\lambda })\Delta u + \Delta p f_u(\hat{w}) +\Delta p f_{uw}(\hat{w})\Delta w \\{} & {} \quad + \Delta \lambda G'(\hat{u}) +\Delta \lambda G''(\hat{u})\Delta u +o(|\Delta w|)=\rho . \end{aligned}$$

Multiplying this relation by $\Delta u$, we get

57 $$\begin{aligned}{} & {} \langle H_{ux}(\hat{w},\hat{p})\Delta x, \Delta u\rangle + \langle \bar{H}_{uu}(\hat{w},\hat{p},\hat{\lambda }) \Delta u,\Delta u\rangle + \Delta p f_u(\hat{w})\Delta u +\langle \Delta p f_{uw}(\hat{w})\Delta w,\Delta u\rangle \nonumber \\{} & {} \quad + \Delta \lambda G'(\hat{u}) \Delta u +\langle \Delta \lambda G''(\hat{u})\Delta u, \Delta u\rangle +o(|\Delta w|^2)=\rho \Delta u. \end{aligned}$$

Adding equalities (56) and (57), we get

$$\begin{aligned}{} & {} \Delta \dot{p}\,\Delta x +\langle H_{xw}(\hat{w},\hat{p})\Delta w, \Delta x\rangle + \langle H_{ux}(\hat{w},\hat{p})\Delta x, \Delta u\rangle + \langle \bar{H}_{uu}(\hat{w},\hat{p},\hat{\lambda }) \Delta u,\Delta u\rangle \\{} & {} \quad +\Delta pf_x(\hat{w})\Delta x +\langle \Delta pf_{xw}(\hat{w}) \Delta w,\Delta x\rangle + \Delta p f_u(\hat{w})\Delta u +\langle \Delta p f_{uw}(\hat{w})\Delta w,\Delta u\rangle \\{} & {} \quad + \Delta \lambda G'(\hat{u}) \Delta u +\langle \Delta \lambda G''(\hat{u})\Delta u, \Delta u\rangle +o(|\Delta w|^2) = \pi \Delta x + \rho \Delta u. \end{aligned}$$

Further, we have

$$\begin{aligned}{} & {} \langle H_{xw}(\hat{w},\hat{p})\Delta w, \Delta x\rangle + \langle H_{ux}(\hat{w},\hat{p})\Delta x, \Delta u \rangle + \langle \bar{H}_{uu}(\hat{w},\hat{p},\hat{\lambda })\Delta u,\Delta u\rangle \\{} & {} \quad =\langle \bar{H}_{xw}(\hat{w},\hat{p},\hat{\lambda })\Delta w, \Delta x\rangle +\langle \bar{H}_{uw}(\hat{w},\hat{p},\hat{\lambda })\Delta w, \Delta u\rangle = \langle \bar{H}_{ww}(\hat{w},\hat{p},\hat{\lambda })\Delta w, \Delta w\rangle . \end{aligned}$$

Moreover,

$$\begin{aligned}{} & {} \Delta pf_x(\hat{w})\Delta x +\langle \Delta pf_{xw}(\hat{w}) \Delta w,\Delta x\rangle + \Delta p f_u(\hat{w})\Delta u +\langle \Delta p f_{uw}(\hat{w})\Delta w,\Delta u\rangle \\{} & {} \quad = \Delta p{f'(\hat{w})}\Delta w+ \langle \Delta p f''(\hat{w})\Delta w,\Delta w\rangle . \end{aligned}$$

Consequently,

$$\begin{aligned}{} & {} \Delta \dot{p}\,\Delta x + \langle \bar{H}_{ww}(\hat{w},\hat{p},\hat{\lambda })\Delta w, \Delta w\rangle + \Delta p {f'(\hat{w})}\Delta w+ \langle \Delta p f''(\hat{w})\Delta w,\Delta w \rangle \\{} & {} \quad +\Delta \lambda G'(\hat{u}) \Delta u +\langle \Delta \lambda G''(\hat{u})\Delta u, \Delta u\rangle +o(|\Delta w|^2) = \pi \Delta x + \rho \Delta u. \end{aligned}$$

Integrating this equality over the segment [0,1], we obtain

$$\begin{aligned}{} & {} \int _0^1 \Delta \dot{p}\,\Delta x\mathrm{\,d}t +\int _0^1\langle \bar{H}_{ww}(\hat{w},\hat{p},\hat{\lambda })\Delta w, \Delta w\rangle \mathrm{\,d}t + \int _0^1\Delta p {f'(\hat{w})}\Delta w \mathrm{\,d}t \\{} & {} \quad + \int _0^1 \langle \Delta p f''(\hat{w})\Delta w,\Delta w \rangle \mathrm{\,d}t +\int _0^1\Delta \lambda G'(\hat{u}) \Delta u \mathrm{\,d}t \\{} & {} \quad +\int _0^1\langle \Delta \lambda G''(\hat{u})\Delta u, \Delta u\rangle \mathrm{\,d}t +\int _0^1 o(|\Delta w|^2)\mathrm{\,d}t = \int _0^1 \left( \pi \Delta x + \rho \Delta u\right) \mathrm{\,d}t. \end{aligned}$$

Integrating by parts the first integral on the left side of this equality and applying (43), we get

$$\begin{aligned}{} & {} \int _0^1 \Delta \dot{p}\,\Delta x\mathrm{\,d}t= \Delta p\,\Delta x\mid _0^1- \int _0^1 \Delta p\,\Delta \dot{x}\mathrm{\,d}t \\{} & {} \quad = \langle F''(\hat{q}) \Delta q,\Delta q\rangle + o( |\Delta q|^2)+\nu \Delta q - \int _0^1 \Delta p\,\Delta \dot{x}\mathrm{\,d}t. \end{aligned}$$

Substituting this expression into the previous equality and taking into account definition (13) of $\Omega$, we get

58 $$\begin{aligned}{} & {} \Omega (\Delta w) + o( |\Delta q|^2)+\nu \Delta q + \int _0^1\Delta p\,\big ( {f'(\hat{w})}\Delta w -\Delta \dot{x} \big )\mathrm{\,d}t\nonumber \\{} & {} \quad + \int _0^1 \langle \Delta p f''(\hat{w})\Delta w,\Delta w\rangle \mathrm{\,d}t +\int _0^1\Delta \lambda G'(\hat{u}) \Delta u \mathrm{\,d}t +\int _0^1\langle \Delta \lambda G''(\hat{u})\Delta u, \Delta u\rangle \mathrm{\,d}t \nonumber \\{} & {} \quad +\int _0^1 o(|\Delta w|^2)\mathrm{\,d}t = \int _0^1 \big (\pi \Delta x + \rho \Delta u\big )\mathrm{\,d}t. \end{aligned}$$

Notice that

$$\begin{aligned} o( |\Delta q|^2)+ \int _0^1 o(|\Delta w|^2)\mathrm{\,d}t=o(\gamma (\Delta w)). \end{aligned}$$

Using this equality and equality (40) in equality (58), we obtain

59 $$\begin{aligned}{} & {} \Omega (\Delta w)+ \nu \Delta q - \int _0^1 \Delta p\,O(|\Delta w|^2)\mathrm{\,d}t+ \int _0^1 \Delta p\,\xi \mathrm{\,d}t\nonumber \\{} & {} \qquad + \int _0^1 \langle \Delta p f''(\hat{w})\Delta w,\Delta w\rangle \mathrm{\,d}t\nonumber \\{} & {} \qquad +\int _0^1\Delta \lambda G'(\hat{u}) \Delta u \mathrm{\,d}t +\int _0^1\langle \Delta \lambda G''(\hat{u})\Delta u, \Delta u \rangle \mathrm{\,d}t +o(\gamma (\Delta w)) \nonumber \\{} & {} \quad = \int _0^1 \big (\pi \Delta x + \rho \Delta u\big )\mathrm{\,d}t. \end{aligned}$$

According to (47), we have $\Vert \Delta p\Vert _\infty \le 2C\delta$. Therefore,

60 $$\begin{aligned} \left| \int _0^1 \Delta p\,O(|\Delta w|^2)\mathrm{\,d}t \right| \le \Vert \Delta p\Vert _\infty \int _0^1|O(|\Delta w|^2)|\mathrm{\,d}t \le c\delta \gamma (\Delta w) \end{aligned}$$

with some $c>0$. Similarly,

61 $$\begin{aligned} \left| \int _0^1 \langle \Delta p f''(\hat{w})\Delta w,\Delta w\rangle \mathrm{\,d}t \right| \le c\delta \gamma (\Delta w). \end{aligned}$$

In addition, in view of (54),

62 $$\begin{aligned} \left| \int _0^1\langle \Delta \lambda G''(\hat{u})\Delta u, \Delta u\rangle \mathrm{\,d}t \right| \le c\delta \gamma (\Delta w) \end{aligned}$$

with some $c>0$. Hence, (59) gives

63 $$\begin{aligned} \Omega (\Delta w)\le & {} -\int _0^1\Delta \lambda G'(\hat{u}) \Delta u \mathrm{\,d}t \nonumber \\{} & {} + \int _0^1 \big (-\Delta p\,\xi +\pi \Delta x + \rho \Delta u\big )\mathrm{\,d}t - \nu \Delta q +C\delta \gamma (\Delta w) \end{aligned}$$

with some $$C>0$$.

**8.** Now we estimate the first term

$$\begin{aligned} -\int _0^1\Delta \lambda G'(\hat{u}) \Delta u \mathrm{\,d}t=-\sum _{j=1}^k \int _0^1\Delta \lambda _j G'_j(\hat{u}) \Delta u \mathrm{\,d}t \end{aligned}$$

in the righ-handt side of inequality (63). Let us fix $j\in \{1,\ldots ,k\}$ and consider the term

$$\begin{aligned} -\int _0^1\Delta \lambda _j G'_j(\hat{u}) \Delta u \mathrm{\,d}t. \end{aligned}$$

We use conditions (4), (9), (27), and (32). If $\Delta \lambda _j=0$, then this term is equal to zero. Therefore, we assume that the set

$$\begin{aligned} M(\Delta \lambda _j) =\{t\in [0,1]\,:\quad \Delta \lambda _j(t)\ne 0 \} \end{aligned}$$

has a positive Lebesgue measure.

8.1. Consider the set

$$\begin{aligned} \{t\in M(\Delta \lambda _j)\,: \lambda _j(t) =0 \}. \end{aligned}$$

A.e. on this set we have

$$\begin{aligned} \Delta \lambda _j=-\hat{\lambda }_j<0. \end{aligned}$$

Then, by the complementary slackness condition in (4), $G_j(\hat{u})=0$. In this case, the condition $G_j(u)\le \eta _j$ yields $G'_j(\hat{u})\Delta u +O(|\Delta u|^2)\le \eta _j$, whence, multiplying by $-\Delta \lambda _j>0$, we get

64 $$\begin{aligned} -\Delta \lambda _j G'_j(\hat{u})\Delta u -\Delta \lambda _j\, O(|\Delta u|^2)\le -\Delta \lambda _j\cdot \eta _j. \end{aligned}$$

8.2. Consider the set

$$\begin{aligned} \{t\in M(\Delta \lambda _j)\,:\; \lambda _j(t) >0 \}. \end{aligned}$$

Then, by the complementary slackness condition in (27), a.e. on this set we have

$$\begin{aligned} G_j(u)=\eta _j. \end{aligned}$$

(a) Let also $G_j(\hat{u})=0$. Then

$$\begin{aligned} G'_j(\hat{u})\Delta u +O(|\Delta u|^2)=\eta _j. \end{aligned}$$

Multiplying this equality by $-\Delta \lambda _j$, we get

$$\begin{aligned} -\Delta \lambda _j G'_j(\hat{u})\Delta u -\Delta \lambda _j\cdot O(|\Delta u|^2)=-\Delta \lambda _j\cdot \eta _j. \end{aligned}$$

(b) Let now $G_j(\hat{u})<0$. Then, by the complementary slackness condition in (4), we have $\hat{\lambda }_j=0$, and then $\Delta \lambda _j=\lambda _j>0.$

Again, by the complementary slackness condition (but now in (27)), we have $G_j(u) =\eta _j$, which implies

$$\begin{aligned} G_j(\hat{u})+ G_j'(\hat{u})\Delta u +O(|\Delta u|^2)=\eta _j. \end{aligned}$$

Multiplying this equality by $-\Delta \lambda _j<0$, we get

$$\begin{aligned} -\Delta \lambda _j \cdot G_j(\hat{u})- \Delta \lambda _j \cdot G'_j(\hat{u})\Delta u - \Delta \lambda _j\cdot O(|\Delta u|^2)=-\Delta \lambda _j\cdot \eta _j. \end{aligned}$$

Since $-\Delta \lambda _j \cdot G_j(\hat{u})>0$, we obtain

$$\begin{aligned} -\Delta \lambda _j G'_j(\hat{u})\Delta u -\Delta \lambda _j\cdot O(|\Delta u|^2)<-\Delta \lambda _j\cdot \eta _j. \end{aligned}$$

Consequently, inequality (64) holds a.e. on the set $M(\Delta \lambda _j)$, and then it holds a.e. on [0.1]. This implies that

65 $$\begin{aligned} -\int _0^1\Delta \lambda _j G'_j(\hat{u})\Delta u \mathrm{\,d}t -\int _0^1\Delta \lambda _j\, O(|\Delta u|^2) \mathrm{\,d}t\le - \int _0^1\Delta \lambda _j\cdot \eta _j \mathrm{\,d}t. \end{aligned}$$

Recall that according to (54), $\Vert \Delta \lambda \Vert _\infty \le C\delta$. Therefore,

$$\begin{aligned} \int _0^1 |\Delta \lambda _j|\cdot |O(|\Delta u|^2)|\mathrm{\,d}t \le C'\delta \,\cdot {\gamma (\Delta w)} \end{aligned}$$

with some $C'>0$. This and (65) imply

$$\begin{aligned} -\int _0^1\Delta \lambda _j G_j'(\hat{u}) \Delta u \mathrm{\,d}t \le - \int _0^1 \Delta \lambda _j\cdot \eta _j\mathrm{\,d}t+C'\delta {\gamma (\Delta w)}. \end{aligned}$$

If $\Delta \lambda _j=0$, then this equality also holds. Thus, it is true for all $j=1,\ldots ,k$. Consequently,

$$\begin{aligned} -\int _0^1\Delta \lambda G'(\hat{u}) \Delta u \mathrm{\,d}t \le \int _0^1 |\Delta \lambda |\cdot |\eta |\mathrm{\,d}t+C'\delta {\gamma (\Delta w)}. \end{aligned}$$

This and inequality (63) imply

66 $$\begin{aligned} \Omega (\Delta w)\le \int _0^1 |\Delta \lambda |\cdot |\eta |\mathrm{\,d}t + \int _0^1 \big (-\Delta p\,\xi +\pi \Delta x + \rho \Delta u\big )\mathrm{\,d}t - \nu \Delta q +c\,\delta \, \gamma (\Delta w)\nonumber \\ \end{aligned}$$

with some $c>0$. Using now the inequality $\Vert \eta \Vert _2\le \Vert \omega \Vert$, we obtain from this that

67 $$\begin{aligned} \Omega (\Delta w)\le \Vert \Delta \lambda \Vert _2\Vert \omega \Vert + \int _0^1 \big (-\Delta p\,\xi +\pi \Delta x + \rho \Delta u\big )\mathrm{\,d}t -\nu \Delta q +c\,\delta \, \gamma (\Delta w).\nonumber \\ \end{aligned}$$

**9.** Let $\Delta >0$ appearing in Assumption 3.1 be given. In order to apply this assumption, with the help of (31) and (32), we pass from the element $\Delta w$ to an element $\delta w\in K_\Delta$, using a "small correction" $w' =\delta w-\Delta w$.

First we use the condition $G(u)\le \eta .$ Let $j\in \{1,\ldots ,k\}$. We remind the notations $M_j := \{t \in [0,1] \, :\;G_j(\hat{u}(t) = 0 \}$ and $M_\Delta ^+(\hat{\lambda }_j) := \{ t \in [0,1] \, :\;\hat{\lambda }_j(t) > \Delta \}$ used in the definition (12) of the cone $K_\Delta$. Set

$$\begin{aligned} M_\Delta (\hat{\lambda }_j)=\{ t\in M_j:\;\hat{\lambda }_j\le \Delta \}. \end{aligned}$$

Then

$$\begin{aligned} M_j=M_\Delta (\hat{\lambda }_j)\cup M_\Delta ^+(\hat{\lambda }_j). \end{aligned}$$

Since $G_j(u)\le \eta _j$ and $G_j(\hat{u})=0$ a.e. on $M_j$, and since $M_\Delta (\hat{\lambda }_j)\subset M_j$, we obtain that

68 $$\begin{aligned} G'_j(\hat{u})\Delta u\le \eta _j-O(|\Delta u|^2)\quad \hbox {a.e. on} \quad M_\Delta (\hat{\lambda }_j). \end{aligned}$$

Now we use the complementary slackness condition in (27). According to this condition, we have $\lambda _j (G_j(u) -\eta _j)=0.$ Using (54), we get

$$\begin{aligned} \lambda _j=\hat{\lambda }_j +\Delta \lambda _j\ge \Delta - |\Delta \lambda _j|\ge \Delta -C\,\delta >0 \quad \hbox {a.e. on} \quad M_\Delta ^+(\hat{\lambda }_j), \end{aligned}$$

whenever $C\,\delta <\Delta .$ Let $\delta >0$ be so small that this condition is fulfilled. Then, it follows that $G_j(u) =\eta _j$ a.e. on $M^+_\Delta (\hat{\lambda }_j)$. Since $G_j(\hat{u})=0$ on $M_j$, we get

69 $$\begin{aligned} G'_j(\hat{u})\Delta u= \eta _j-O(|\Delta u|^2)\quad \hbox {a.e. on} \quad M_\Delta ^+(\hat{\lambda }_j). \end{aligned}$$

By virtue of Assumption 2.1, relations (68) and (69) imply that there exists $$ u'$$ such that for all $$j\in \{1,\ldots ,k\}$$ we have

70 $$\begin{aligned}{} & {} G_j'(\hat{u})\big (\Delta u+ u'\big )\le 0 \quad \hbox {a.e. on} \quad M_\Delta (\hat{\lambda }_j), \end{aligned}$$

71 $$\begin{aligned}{} & {} G'_j(\hat{u})\big (\Delta u+ u'\big )=0, \quad \hbox {a.e. on} \quad M^+_\Delta (\hat{\lambda }_j), \end{aligned}$$

72 $$\begin{aligned}{} & {} |u'|\le c\big (|\eta |+O(|\Delta u|^2)\big ) \end{aligned}$$

with some $c>0$, and, therefore,

73 $$\begin{aligned} \Vert u'\Vert _1\le c\Vert \eta \Vert _1+O(\Vert \Delta u\Vert _2 ^2)\le c\Vert \omega \Vert +O(\Vert \Delta u\Vert _2 ^2). \end{aligned}$$

Here we use $\Vert \eta \Vert _1\le \Vert \eta \Vert _2\le \Vert \omega \Vert$. Moreover, due to (72) and since $\Vert \Delta u\Vert _\infty \le \delta$, the product of functions $|\Delta u|\cdot |u'|$ satisfies the estimate

74 $$\begin{aligned} \int _0^1|\Delta u|\cdot |u'|\mathrm{\,d}t\le c\Vert \Delta u \Vert _2 \Vert \omega \Vert + c'\delta \Vert \Delta u\Vert _2 ^2 \end{aligned}$$

with some $c'>0$, and also by virtue of (72) for the function $| u'|^2$ we have the estimate

75 $$\begin{aligned} \int _0^1|u'|^2\mathrm{\,d}t =\Vert u' \Vert _2^2\le 2c^2\Vert \eta \Vert _2^2+ c'\int _0^1|\Delta u |^4\mathrm{\,d}t\le c\Vert \omega \Vert ^2+ c'\delta ^2\Vert \Delta u\Vert _2 ^2 \end{aligned}$$

with some $c>0$ and $c'>0$.

**10.** Set

$$\begin{aligned} \delta u=\Delta u+u'. \end{aligned}$$

There exists $\delta x\in W^{1,1}$ such that

76 $$\begin{aligned} \delta \dot{x}=f_x(\hat{w})\delta x+ f_u(\hat{w})\delta u,\quad \delta x(0)=\Delta x(0). \end{aligned}$$

Recall that by (40)

$$\begin{aligned} \Delta \dot{x}=f_x(\hat{w})\Delta x+ f_u(\hat{w})\Delta u+O(|\Delta w|^2)-\xi . \end{aligned}$$

Then $$\delta x= \Delta x+ x'$$, where $$ x'$$ satisfies

$$\begin{aligned} \dot{ x}'=f_x(\hat{w}) x' + f_u(\hat{w}) u'-O(|\Delta w|^2)+\xi ,\quad x'(0)=0. \end{aligned}$$

This and (73) imply the following estimate

77 $$\begin{aligned} \Vert x'\Vert _\infty \le c(\Vert u'\Vert _1+\Vert \xi \Vert _1)+O(\Vert \Delta w\Vert _2^2)\le c'\Vert \omega \Vert + O(\Vert \Delta w\Vert _2^2) \end{aligned}$$

with some $$c>0$$ and $$c'>0$$. Set $$ w'=( x', u')$$. Then $$\delta w=\Delta w+ w'$$. Due to (70) and (71), it is easy to verify that

$$\begin{aligned} \delta w=(\delta x,\delta u)\in K_\Delta , \end{aligned}$$

and hence, by Assumption 3.1 (see also Remark 3.1),

78 $$\begin{aligned} \Omega (\delta w)\ge c_\Delta \gamma (\delta w). \end{aligned}$$

**11.** Let us compare $\Omega (\delta w)$ with $\Omega (\Delta w)$. According to Lemma 4.1, we have

79 $$\begin{aligned} \Omega (\delta w) = {\Omega (\Delta w + w') } = \Omega (\Delta w)+ E(\Delta w,w'), \end{aligned}$$

where

80 $$\begin{aligned}{} & {} |E(\Delta w,w')|\le c_E\big ( \Vert \Delta x\Vert _\infty \Vert x'\Vert _\infty +\Vert x'\Vert _\infty ^2 +\Vert x'\Vert _\infty \Vert \Delta u\Vert _1\nonumber \\{} & {} \quad +\Vert \Delta x\Vert _\infty \Vert u'\Vert _1 +\Vert x'\Vert _\infty \Vert u'\Vert _1 +\Vert u'\Vert _2^2 + \Vert |\Delta u| \cdot |u'|\Vert _1\big ). \end{aligned}$$

According to the above estimates (72)-(75), and (77) (we replace $c'$ with *c*, taking the maximum of these two constants as the new *c*), we have

$$\begin{aligned}{} & {} \Vert \Delta x\Vert _\infty \Vert x'\Vert _\infty \le c\Vert \Delta x\Vert _\infty \Vert \omega \Vert + o(\gamma (\Delta w)), \\{} & {} \Vert x'\Vert _\infty ^2\le \big (c\Vert \omega \Vert + O(\Vert \Delta w\Vert _2^2)\big )^2\le 2c^2\Vert \omega \Vert ^2 + 2 O(\Vert \Delta w\Vert _2^4)\le 2c^2\Vert \omega \Vert ^2 + o(\gamma (\Delta w)) , \\{} & {} \Vert \Delta u\Vert _1 \Vert x'\Vert _\infty \le \Vert \Delta u\Vert _2 \Vert x'\Vert _\infty \le c\Vert \Delta u\Vert _2 \Vert \omega \Vert + o(\gamma (\Delta w)), \\{} & {} \Vert \Delta x\Vert _\infty \Vert u'\Vert _1\le c\Vert \Delta x\Vert _\infty \Vert \omega \Vert + o(\gamma (\Delta w)), \\{} & {} \Vert x'\Vert _\infty \Vert u'\Vert _1\le \big (c\Vert \omega \Vert + O(\gamma (\Delta w)\big )^2\le 2c^2 \Vert \omega \Vert ^2+ o(\gamma (\Delta w)), \\{} & {} \Vert u'\Vert _2^2\le c\Vert \omega \Vert ^2+ c\delta ^2\Vert \Delta u\Vert _2 ^2, \\{} & {} \Vert |\Delta u|\cdot |u'|\Vert _1\le c\Vert \omega \Vert \Vert \Delta u \Vert _2+ c\delta \Vert \Delta u\Vert _2 ^2. \end{aligned}$$

This implies that

81 $$\begin{aligned} |E(\Delta w,w')|\le c_\Omega R_\delta (\Delta w,\omega ) \end{aligned}$$

with some $c_\Omega > 0$, where (provided that $\delta > 0$ is sufficiently small)

$$\begin{aligned} \, R_\delta (\Delta w,\omega ):=\Vert \omega \Vert ^2+ \Vert \omega \Vert \Vert \Delta x\Vert _\infty + \Vert \omega \Vert \Vert \Delta u \Vert _2+ \delta \gamma (\Delta w). \end{aligned}$$

**12.** Let us compare $\gamma (\delta w)$ with $\gamma (\Delta w)$. We have

82 $$\begin{aligned} \gamma (\delta w)= \gamma (\Delta w)+ r_\gamma (\Delta w,w'), \end{aligned}$$

where

$$\begin{aligned} r_\gamma (\Delta w,w'):=\Vert \Delta x+x'\Vert _\infty ^2-\Vert \Delta x\Vert _\infty ^2 +2\int _0^1\langle \Delta u,u' \rangle \mathrm{\,d}t + \int _0^1\langle u',u' \rangle \mathrm{\,d}t. \end{aligned}$$

Here

$$\begin{aligned}{} & {} \big |{\Vert \Delta x+x'\Vert ^2_\infty }-\Vert \Delta x\Vert _\infty ^2\big | =\big |\Vert \Delta x+x'\Vert _\infty -\Vert \Delta x\Vert _\infty \big |\cdot \big |\Vert \Delta x+x'\Vert _\infty +\Vert \Delta x\Vert _\infty \big |\} \\{} & {} \quad { \le c\Vert x'\Vert _\infty \big ( 2\Vert \Delta x\Vert _\infty +\Vert x'\Vert _\infty \big ) } \end{aligned}$$

with some $c>0$. This implies that

$$\begin{aligned} |r_\gamma (\Delta w,w')|\le c_r\big ( \Vert \Delta x\Vert _\infty \Vert x'\Vert _\infty +\Vert x'\Vert _\infty ^2 + \Vert |\Delta u|\cdot |u'|\Vert _1 +\Vert u'\Vert _2^2 \big ) \end{aligned}$$

with some $c_r>0$. All these terms are contained in the estimate (80) for $|E(\Delta w,w')|$. Consequently,

83 $$\begin{aligned} |r_\gamma (\Delta w,w')|\le c_\gamma \, R_\delta (\Delta w,\omega ) \end{aligned}$$

with some $$c_\gamma >0$$.

**13.** Inequality (78) along with relations (79) and (82) implies the inequality

$$\begin{aligned} \Omega (\Delta w)+ E(\Delta w,w')\ge c_\Delta \big (\gamma (\Delta w)+ r_\gamma (\Delta w,w')\big ), \end{aligned}$$

whence

$$\begin{aligned} c_\Delta \gamma (\Delta w)- c_\Delta |r_\gamma (\Delta w,w')| -| E(\Delta w,w')|\le \Omega (\Delta w). \end{aligned}$$

Using estimates (81) and (83) in this inequality, we get

84 $$\begin{aligned} c_\Delta \gamma (\Delta w)- (c_\Delta c_\gamma +c_\Omega )\, R_\delta (\Delta w,\omega )\le \Omega (\Delta w). \end{aligned}$$

**14.** Combining inequality (67) with (84) we get

$$\begin{aligned}{} & {} c_\Delta \gamma (\Delta w)- (c_\Delta c_\gamma +c_\Omega )\, R_\delta (\Delta w,\omega ) \le \Omega (\Delta w) \\{} & {} \quad \le \Vert \Delta \lambda \Vert _2\Vert \omega \Vert + \int _0^1 \big (-\Delta p\,\xi +\pi \Delta x + \rho \Delta u\big )\mathrm{\,d}t -\nu \Delta q +c\,\delta \, \gamma (\Delta w). \end{aligned}$$

Consequently,

$$\begin{aligned}{} & {} c_\Delta \gamma (\Delta w) \le (c_\Delta c_\gamma +c_\Omega )\, R_\delta (\Delta w,\omega )+ \Vert \Delta \lambda \Vert _2\Vert \omega \Vert \\{} & {} \quad +\Vert \Delta p\Vert _\infty \Vert \xi \Vert _1+\Vert \pi \Vert _1\Vert \Delta x\Vert _\infty +\Vert \rho \Vert _2\Vert \Delta u\Vert _2 + |\nu |\cdot |\Delta q| +c\,\delta \, \gamma (\Delta w). \end{aligned}$$

Substituting the expression for $\, R_\delta (\Delta w,\omega )$ in this inequality, we obtain that

$$\begin{aligned}{} & {} c_\Delta \gamma (\Delta w)\le \tilde{c} \Big ( \Vert \omega \Vert ^2+ \Vert \omega \Vert \Vert \Delta x\Vert _\infty + \Vert \omega \Vert \Vert \Delta u \Vert _2+ \delta \gamma (\Delta w)) \Big ) +\Vert \Delta \lambda \Vert _2\Vert \omega \Vert \\{} & {} \quad +\Vert \Delta p\Vert _\infty \Vert \xi \Vert _1+\Vert \pi \Vert _1\Vert \Delta x\Vert _\infty +\Vert \rho \Vert _2\Vert \Delta u\Vert _2 + |\nu |\cdot |\Delta q| +c\,\delta \, \gamma (\Delta w), \end{aligned}$$

where $$\tilde{c}=c_\Delta c_\gamma +c_\Omega $$. Then

$$\begin{aligned}{} & {} (c_\Delta -\tilde{c}\,\delta -c\,\delta )\gamma (\Delta w)\le \tilde{c} \Big ( \Vert \omega \Vert ^2+ \Vert \omega \Vert \Vert \Delta x\Vert _\infty + \Vert \omega \Vert \Vert \Delta u \Vert _2 \Big ) +\Vert \Delta \lambda \Vert _2\Vert \omega \Vert \\{} & {} \quad +\Vert \Delta p\Vert _\infty \Vert \xi \Vert _1+\Vert \pi \Vert _1\Vert \Delta x\Vert _\infty +\Vert \rho \Vert _2\Vert \Delta u\Vert _2 + |\nu |\cdot |\Delta q|. \end{aligned}$$

Take $\delta >0$ so small that $c_\Delta ' := c_\Delta -\tilde{c}\,\delta -c\,\delta >0$. Then

85 $$\begin{aligned}{} & {} c_\Delta '(\Vert \Delta x\Vert _\infty ^2+ \Vert \Delta u\Vert _2^2) \le { \tilde{c}( \Vert \omega \Vert ^2+ \Vert \omega \Vert \Vert \Delta x\Vert _\infty + \Vert \omega \Vert \Vert \Delta u \Vert _2)} +\Vert \Delta \lambda \Vert _2\Vert \omega \Vert \nonumber \\{} & {} \quad +\Vert \Delta p\Vert _\infty \Vert \xi \Vert _1+\Vert \pi \Vert _1\Vert \Delta x\Vert _\infty +\Vert \rho \Vert _2\Vert \Delta u\Vert _2 + |\nu |\cdot |\Delta q|. \end{aligned}$$

Relations (38) and (46) imply

$$\begin{aligned} \Vert \Delta x\Vert _\infty \le C\big (|\Delta x_0|+\Vert \Delta u\Vert _2 +\Vert \omega \Vert \big ), \quad \Vert \Delta p\Vert _\infty \le C\big (|\Delta x_0|+\Vert \Delta u\Vert _2 +\Vert \omega \Vert \big ). \end{aligned}$$

Moreover, according (55), we have

$$\begin{aligned} \Vert \Delta \lambda \Vert _2\le C\big (|\Delta x_0|+\Vert \Delta u\Vert _2 +\Vert \omega \Vert \big ). \end{aligned}$$

Using these relations in (85) together with the definition $\Vert \omega \Vert :=|\nu |+ \Vert \pi \Vert _1+\Vert \rho \Vert _2+ \Vert \xi \Vert _1+\Vert \eta \Vert _2$ and taking into account the inequalities $|\Delta x_0|\le |\Delta q|\le 2 \Vert \Delta x\Vert _\infty$, we get

$$\begin{aligned} c_\Delta ''\big (|\Delta x_0|^2+ \Vert \Delta u\Vert _2^2\big ) \le (|\Delta x_0|+\Vert \Delta u\Vert _2)\Vert \omega \Vert +\Vert \omega \Vert ^2 \end{aligned}$$

with some $c_\Delta ''>0$ provided that $\delta >0$ is small enough. Set $z=|\Delta x_0|+\Vert \Delta u\Vert _2$, $y=\Vert \omega \Vert$. Since $|\Delta x_0|^2+\Vert \Delta u\Vert _2^2\ge \frac{1}{2} z^2$, we obtain

$$\begin{aligned} az^2\le zy+y^2, \end{aligned}$$

where $a=c_\Delta ''/2$. This implies that

$$\begin{aligned} bz\le y, \quad \hbox {where} \quad b=\frac{\sqrt{4a+1}-1}{2}. \end{aligned}$$

Consequently, $b (|\Delta x_0|+\Vert \Delta u\Vert _2)\le \Vert \omega \Vert ,$ or equivalently,

86 $$\begin{aligned} |\Delta x_0|+\Vert \Delta u\Vert _2\le c_1 \Vert \omega \Vert , \end{aligned}$$

where $c_1=1/b$. Then relations (38), (46), and (55) imply

87 $$\begin{aligned} \Vert \Delta x\Vert _{1,1}\le c_2\Vert \omega \Vert ,\quad \Vert \Delta p\Vert _{1,1} \le c_3\Vert \omega \Vert , \quad \Vert \Delta \lambda \Vert _2 \le c_4\Vert \omega \Vert \end{aligned}$$

with some $c_2>0$, $c_3>0$, and $c_4>0$. The theorem is proved.

## Footnotes

1 This means that $J(\hat{x},\hat{u}) \le J(x,u)$ for every admissible pair (*x*, *u*) which is close enough to $(\hat{x}, \hat{u})$ in the space $\mathcal{W}$.
